# The Host Cell Transcription Factor EGR1 Is Induced by Bacteria through the EGFR–ERK1/2 Pathway

**DOI:** 10.3389/fcimb.2017.00016

**Published:** 2017-01-25

**Authors:** Nele de Klerk, Sunil D. Saroj, Gabriela M. Wassing, Lisa Maudsdotter, Ann-Beth Jonsson

**Affiliations:** Department of Molecular Biosciences, The Wenner-Gren Institute, Stockholm UniversityStockholm, Sweden

**Keywords:** EGR1, bacterial signaling, adhesion, infection, early events, EGFR

## Abstract

The essential first step in bacterial colonization is adhesion to the host epithelial cells. The early host-responses post-bacterial adhesions are still poorly understood. Early growth response 1 (EGR1) is an early response transcriptional regulator that can be rapidly induced by various environmental stimuli. Several bacteria can induce EGR1 expression in host cells, but the involved bacterial characteristics and the underlying molecular mechanisms of this response are largely unknown. Here, we show that EGR1 can be induced in host epithelial cells by different species of bacteria independent of the adherence level, Gram-staining type and pathogenicity. However, bacterial viability and contact with host cells is necessary, indicating that an active interaction between bacteria and the host is important. Furthermore, the strongest response is observed in cells originating from the natural site of the infection, suggesting that the EGR1 induction is cell type specific. Finally, we show that EGFR–ERK1/2 and β1-integrin signaling are the main pathways used for bacteria-mediated EGR1 upregulation. In conclusion, the increase of EGR1 expression in epithelial cells is a common stress induced, cell type specific response upon host-bacteria interaction that is mediated by EGFR–ERK1/2 and β1-integrin signaling.

## Introduction

Early growth response 1 (EGR1) is a zinc-finger transcription factor that is also known as Zif268, NGFI-A, Krox24, and TIS8. As a transcriptional regulator, EGR1 plays an important role in the regulation of cell physiology affecting growth, differentiation and survival. EGR1 is ubiquitously expressed in human tissues and can be rapidly induced by a great variety of environmental signals, such as growth factors, shear stress, reactive oxygen species and cytokines (Cao et al., 1992; Sadoshima et al., 1992; Nose and Ohba, 1996; Mayer et al., 2009). Induction of EGR1 can be mediated by several signaling pathways, including PKA and the MAP kinases ERK1/2, JNK and p38 (Pagel and Deindl, 2011). EGR1 in turn recognizes and binds to the DNA consensus sequence GCG(G/T)GGGCG (Swirnoff and Milbrandt, 1995). Thereby, EGR1 can regulate the transcription of many different genes with diverse functions, including cell cycle regulatory proteins, extracellular matrix proteins, transcriptional regulatory proteins, cytokines and growth factors (Krämer et al., 1994; Skerka et al., 1995; Svaren et al., 2000; Fu et al., 2003; Hoffmann et al., 2008; Kerpedjieva et al., 2012). Anomalies in the expression of EGR1 have been implicated in various tissue pathophysiologies, such as carcinogenesis, inflammation and ischemic injury (Pawlinski et al., 2003; Baron et al., 2006; Yang et al., 2015).

Some bacteria can induce EGR1 expression, such as *Chlamydia pneumoniae, Helicobacter pylori, Neisseria meningitidis, Neisseria gonorrhoeae, Porphyromonas gingivalis*, and *Streptococcus intermedius* (Abdel-Latif et al., 2004; Howie et al., 2005; Rupp et al., 2005; Schubert-Unkmeir et al., 2007; Maekawa et al., 2010; Susilowati et al., 2011). Some of these studies have identified ERK as an important signaling molecule, but additional information on the mechanisms underlying bacterial EGR1 induction and its role in virulence is very scarce.

However, for *H. pylori* it has been shown that epidermal growth factor receptor (EGFR) transactivation is partially involved and an intact Cag secretion system is necessary (Keates et al., 2005). For the enterobacteriaceae family members *Salmonella enterica* serovar Typhimurium, *Yersinia pseudotuberculosis*, and enteropathogenic *Escherichia coli* EGR1 induction is type III secretion system dependent (de Grado et al., 2001; Hannemann et al., 2013; Kwuan et al., 2013).

The first step in bacterial pathogenesis is the colonization of the infection site through active adherence of pathogens to specific tissues. Bacterial adherence to the host epithelia generally depicts a receptor-ligand model. The bacterial adhesins act as a ligand that binds to specific receptors on the host epithelia. Colonization may not necessarily result in invasion or an inflammatory response. Host-pathogen interaction is a dynamic phenomenon; additional information about the early events that occur during host-pathogen interaction can provide new insights on bacterial virulence and pathogenicity. Although the role of EGR1 as an immediate early response factor is well established in the regulation of inflammatory and immune responses, there is limited information on whether EGR1 induction is a general response by host cells upon infection by all bacteria or a response specific for a particular bacterial strain. Also, the exact molecular pathway followed by each bacterium to induce EGR1 is not known. Therefore, the current study sought to determine whether bacterial adherence induces EGR1, whether the induction is common or specific to a selected group of bacteria, the molecular mechanisms involved and the role of EGR1 in bacterial adherence. We show that most bacteria can upregulate EGR1 in host epithelial cells, independent of the level of adherence, Gram-staining type and pathogenicity. Moreover, EGR1 upregulation is a cell type specific phenomenon, and is dependent on bacterial viability and host cell contact. Furthermore, the main pathways utilized by bacteria to trigger EGR1 expression are EGFR–ERK1/2 and β1-integrin signaling.

## Materials and methods

### Bacterial strains and culture conditions

All bacterial strains used in this study are listed in Table 1. All *Neisseria* strains and *Streptococcus pyogenes* strains were grown on GC agar (Acumedia) containing Kellogg's supplement (Kellogg et al., 1963). *Pseudomonas aeruginosa, Staphylococcus aureus*, all *Salmonella* strains and the *E. coli* strains were grown on Luria agar (Acumedia). The *Lactobacillus* strains were grown on Rogosa agar (Oxoid). All aforementioned bacteria were cultured at 37°C and 5% CO_2_ for 16–18 h before experimentation. The *Helicobacter pylori* strains were grown on Colombia blood agar (Acumedia) supplemented with 5% defibrinated horse blood and 5% inactivated horse serum (Håtunalab) for 3 days at 37°C under microaerophilic conditions (5% O_2_, 10% CO_2_). Before each experiment, the bacteria were washed once and resuspended in cell culture medium without serum that was specific to the cell line that was used.

**Table 1:** **Bacterial strains used in this study**.

	**PATHOGEN**		**NON-PATHOGEN**	
**Colonization Site (cell line)**	**Abbreviation**	**Strain**	**Abbreviation**	**Strain**
Upper respiratory tract (FaDu)	Nm-A	*Neisseria menigitidis* serogroup A Z2491	Ns	*Neisseria subflava* GN01 (Jonsson et al., 1991)
	Nm-B	*Neisseria menigitidis* serogroup B MC58	Nl	*Neisseria lactamica* NCTC 10618 (Jonsson et al., 1991)
	Nm-C	*Neisseria menigitidis* serogroup C FAM20 (Rahman et al., 1997)		
	Nm-W	*Neisseria menigitidis* serogroup W-135 JB515 (Rahman et al., 1997)		
	Pa	*Pseudomonas aeruginosa* PAO1		
	Sp-M1	*Streptococcus pyogenes* serogroup M1 S340	Lr	*Lactobacillus reuteri* ATCC PTA5289
	Sp-M3	*Streptococcus pyogenes* serogroup M3 S208	Ls	*Lactobacillus salivarius* LMG9477
	Sp-M5	*Streptococcus pyogenes* serogroup M5 Manfredo (Johnsson et al., 1998)		
	Sp-M6	*Streptococcus pyogenes* serogroup M6 S165 (Sjölinder et al., 2008)		
	Sa	*Staphylococcus aureus* Newman		
Stomach (AGS)	Hp-J99	*Helicobacter pylori* J99 (ATCC 700824)	Lrh	*Lactobacillus rhamnosus* Kx151A1 (Roos et al., 2005)
	Hp-6721	*Helicobacter pylori* 67:21 (Björkholm et al., 2001)		
Intestine (Caco-2)	Ec-B09	*Escherichia coli* B09-11822 (Skorup et al., 2014)	Lrh-GG	*Lactobacillus rhamnosus* GG (ATCC 53103)
	Ec-O11	*Escherichia coli* O111:B4	Ec-DH5α	*Escherichia coli* DH5α
	SE-3934	*Salmonella enterica* serovar Enteritidis 3934		
	STM-42	*Salmonella enterica* serovar Typhimurium FIA42		
Urogenital tract (ME180)	Ng	*Neisseria gonorrhoeae* MS11 (ATCC BAA1833)	Lc	*Lactobacillus crispatus* MV24-1a

### Cell lines and culture conditions

The human pharyngeal epithelial cell line FaDu (ATCC HTB-43), the human colon epithelial cell line Caco-2 (ATCC HTB-37) and the human cervical epithelial cell line ME180 (ATCC HTB-33) were cultured in DMEM + GlutaMAX (Invitrogen) supplemented with 10% heat-inactivated fetal bovine serum (Sigma Aldrich). The human gastric epithelial cell line AGS (ATCC CRL-1739) was cultured in RPMI-1640 + GlutaMAX (Invitrogen) supplemented with 10% heat-inactivated fetal bovine serum. All cell lines were maintained at 37°C and 5% CO_2_. The cells were seeded into 24-well tissue culture plates the day before the experiment to form a monolayer overnight. Before each experiment, the cells were washed twice with cell culture medium without serum.

### qPCR analysis

The epithelial cells were infected with bacteria to a multiplicity of infection (MOI) of 100. The bacteria were not removed after addition to the epithelial cells. In some experiments Millicell 0.4 μm filters (Millipore) were used to prevent direct bacterial contact to the host epithelial cells. For infection with dead bacteria, a dense suspension of bacteria was heat-killed at 95°C for 10 min and diluted in cell culture medium to the desired density for infection. After 1, 2, 4, and 6 h of incubation RNA was isolated using the RNeasy Plus kit (Qiagen) according to manufacturer's instructions. The concentration of the RNA was determined using the NanoDrop 8000 (Thermo Scientific) UV-Vis Spectrophotometer. Total RNA was reverse transcribed to cDNA using SuperScript VILO Mastermix (Invitrogen). Quantitative PCR was performed using a LightCycler 480 (Roche) and the SYBRGreen I Master kit (Roche). The primers used are listed in Table 2. The thermal cycling conditions were: initial denaturation at 95°C for 10 min followed by amplification for 40 cycles with denaturation at 95°C for 10 s, annealing at 50°C for 20 s and extension at 72°C for 20 s. The melting curve analysis was as follows: 95°C for 5 s, 65°C for 1 min and then increasing to 95°C at 0.08°C/s. The expression was normalized against the housekeeping gene β-actin. The expression levels were calculated by the comparative C_T_ method (ΔΔ C_T_ method) expressed as the fold change compared to uninfected cells.

**Table 2:** **Primers used in this study**.

**Gene**	**Primer**	**Sequence (5′-3′)**	**References**
β-Actin	bAct F	CATGCCATCCTGCGTCTGGACC	Wex et al., 2004
	bAct R	ACATGGTGGTGCCGCCAGACAG	
EGR1	EGR1 F	CCCGTTCGGATCCTTTCCT	Keates et al., 2005
	EGR1 R	CAGCATCATCTCCTCCAGCTT	
β1-integrin	ITGβ1 F	GAAGGGTTGCCCTCCAGA	Dingemans et al., 2010
	ITGβ1 R	GCTTGAGCTTCTCTGCTGTT	
Firbronectin	Fn F	GGAGTTGATTATACCATCACTG	Tang et al., 2010
	Fn R	TTTCTGTTTGATCTGGACCT	
Amphiregulin	Amph F	GTGGTGCTGTCGCTCTTGATACTC	Löfmark et al., 2011
	Amph R	TCAAATCCATCAGCACTGTGGTC	
EGFR	EGFR F	ACTGCACCTACGGATGCACTGG	Löfmark et al., 2011
	EGFR R	AACGATGTGGCGCCTTCGCA	
ILK	ILK2-F	GGGCTCTTGTGAGCTTCTGT	Cano-Peñalver et al., 2014
	ILK2-R	GAGTGGTCCCCTTCCAGAAT	

### Western blot analysis

The epithelial cells were infected with bacteria to a MOI of 100. The bacteria were not removed after addition to the epithelial cells. After incubation for the indicated time points the cells were washed twice with PBS and directly placed on ice. The cells were lysed with 50 μl of 1X sample buffer (63 mM Tris-HCl pH 6.8, 25% glycerol, 1% SDS, 5% 2-mercaptoethanol), boiled for 10 min at 95°C and stored at −20°C until use. Thawed samples were centrifuged for 1 min at 10,000 × g and 15 μl was loaded on 10% acrylamide SDS-PAGE gels. After separation, the proteins were transferred to Immobilon-P PVDF membranes (Millipore) using a semi-dry transfer system (Bio-Rad). The membranes were washed in water and blocked for 1 h in 5% skim milk powder (Sigma Aldrich) in PBS at room temperature. The membranes were incubated overnight at 4°C with antibodies against EGR1 (Abcam, ab194357, 1:10,000 dilution) and β-actin (Millipore, MAB1501, 1:2000 dilution) in 1% skim milk powder in PBS. After washing 3 times with PBS, the membranes were incubated with IRDye800-conjugated goat-anti-rabbit and IRDye680-conjugated goat-anti-mouse antibodies (LI-COR, 1:10,000 dilution) for 1 h at room temperature. Bands were visualized using an Odyssey IR scanner (LI-COR).

### Adhesion assays

The epithelial cells were infected with bacteria to a MOI of 100. The bacteria were not removed after addition to the epithelial cells. After incubation, the cells were washed three times with PBS to remove unbound bacteria. Bacterial adherence was estimated from viable counts by lysing the host epithelial cells with 1% saponin in cell culture medium for 10 min and plating serial dilutions. Viable counts for *P. aeruginosa* were performed on Luria agar plates, incubated at 37°C and 5% CO_2_ and the colony forming units (cfu) were counted the following day. Viable counts for *H. pylori* were performed on blood agar plates incubated at 37°C under microaerophilic conditions for 4–7 days. Viable counts for all other bacteria were performed on GC agar and incubated at 37°C and 5% CO_2_ for 1–2 days.

### Chemical inhibition of signaling pathways

PD153035 (125 nM), PD184352 (125 nM), SP600125 (25 μM), p38 MAP kinase inhibitor IV (1 μM) and Protein Kinase A inhibitor fragment 14:22 (10 μM) are chemical inhibitors of the EGFR, ERK1/2, JNK, p38 and PKA signaling molecules, respectively. All inhibitors used were purchased from Sigma Aldrich and resuspended in DMSO. The cells were pre-treated with the inhibitors for 1 h and then co-incubated with the inhibitors and bacteria (MOI 100) for 2 h at 37°C and 5% CO_2_. In the experiments with *N. gonorrhoeae* the cells were co-incubated for 4 h. Following incubation, adhesion assays and qPCR analysis was performed as described in the sections above.

### RNA silencing

The epithelial cells were seeded into 24-well tissue culture plates to a confluency of 50–80% prior to transfection. For RNA silencing, AGS cells were replaced by MKN-45. The cells were washed twice with serum-free cell culture medium and transfected with 25 nM ON-Target Plus SMARTpool siRNA (Dharmacon) in Opti-MEM (Invitrogen) using Lipofectamine RNAiMAX (Invitrogen) according to manufacturer's recommendations. Following an overnight incubation cell culture medium supplemented with 10% FBS was added. The cells were maintained for a further 48 h at 37°C and 5% CO_2_ before use in experiments for bacterial adhesion and qPCR analysis as described in the sections above. The efficiency of knockdown was determined using qPCR.

### Statistical analysis

All experiments were performed in triplicate and repeated three times. Analysis of variance (ANOVA) and the Student's *t*-test were employed to analyze the difference between the groups for statistical significance. *P* < 0.05 was considered statistically significant. The data is represented as the mean ± standard deviation. The asterisk in the bar graph denote statistical significance. The significance level is represented in the graphs as ^***^P < 0.001, ^**^P < 0.01, ^*^P < 0.05, NS-non significant.

## Results

### Bacteria induce EGR1 expression in host epithelial cells

**Figure 1:**
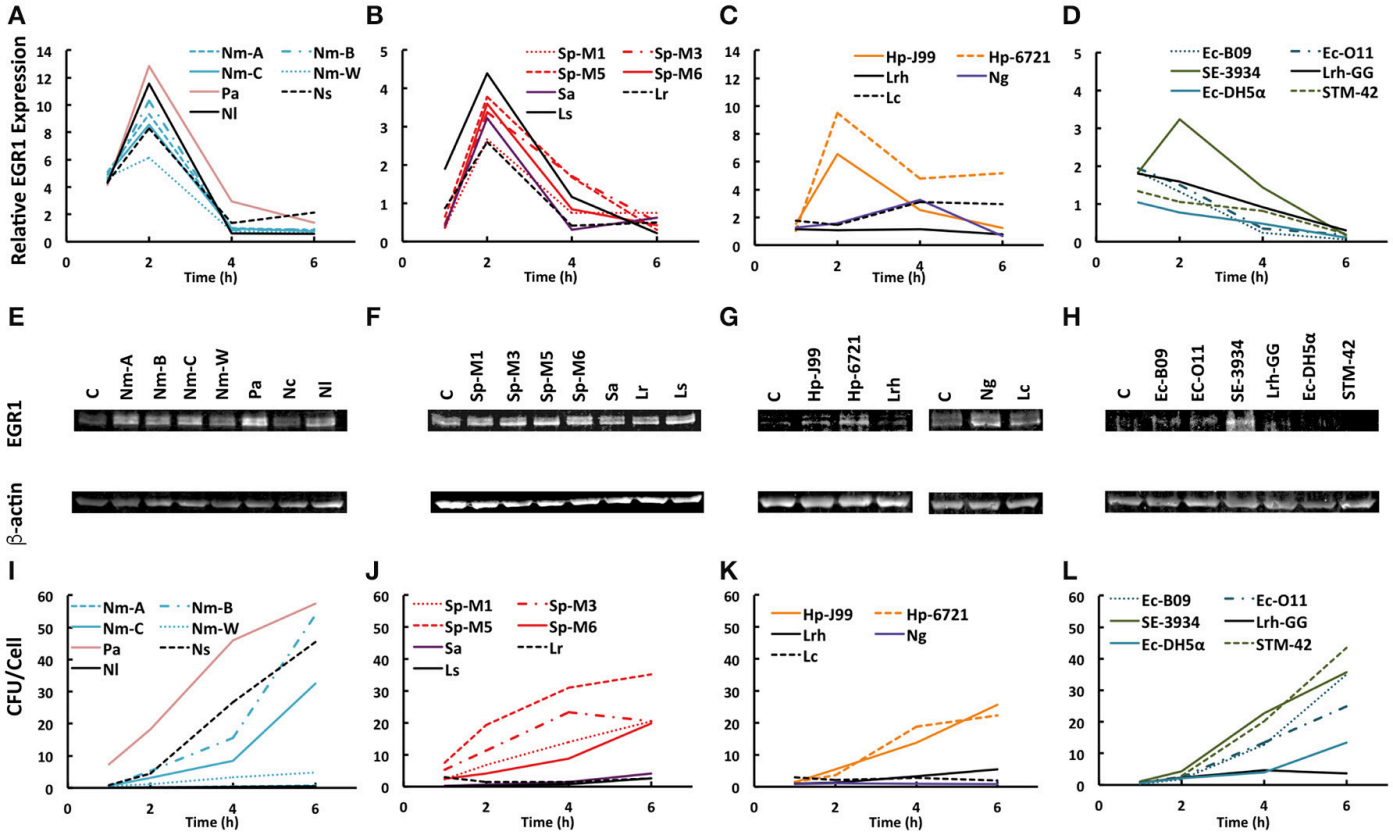
**Bacteria mediated EGR1 induction in host epithelial cells. (A–D)** EGR1 expression was evaluated using qPCR at 1, 2, 4, and 6 h after the addition of bacteria at a multiplicity of infection (MOI) = 100. The data is represented as fold change relative to uninfected. **(E–H)** EGR1 expression evaluated using Western blotting. The blots represent the 4 h time point during the course of infection. β-actin expression was used as a loading control. **(I–L)** Bacterial attachment to cells at different time points after bacterial inoculation (MOI = 100). Bacteria were diluted and plated for viable counts to determine colony forming units (CFU)/ml. **(A,E,I)** Pharyngeal FaDu cells were inoculated with different strains of *N. meningitidis* (Nm-A, Nm-B, Nm-C, Nm-W), *P. aeruginosa* (Pa), *N. subflava* (Ns), and *N. lactamica*. **(B,F,J)** FaDu cells were inoculated with *S. pyogenes* (Sp-M1, Sp-M3, Sp-M5, Sp-M6), *S. aureus* (Sa), *L. reuteri* (Lr), and *L. salivarius* (C, G, K) Gastric AGS cells were inoculated with *H. pylori* (Hp-J99, Hp-6721) and *L. rhamnosus* (Lrh). Cervical ME-180 cells were infected with *N. gonorrhoeae* (Ng) and *L. crispatus* (Lc). **(D,H,L)** Intestinal Caco-2 cells were inoculated with *E. coli* (Ec-B09, Ec-O11, Ec-DH5α), *L. rhamnosus* GG (Lrh-GG), *Salmonella enterica* serovar *Enteritidis* (SE-3934) and *S. enterica* serovar Typhimurium (STM-42).

Only a few studies have investigated the bacterial induction of EGR1 in host cells. To determine whether the induction of EGR1 is a general stress response of the host cells to bacterial colonization, we performed a screen using 25 different bacterial strains including both Gram-positive and Gram-negative pathogens and non-pathogens. We divided the bacterial strains into groups depending on the site of isolation. Bacteria isolated from the upper respiratory tract were added to FaDu pharyngeal epithelial cells (Figures 1A,B), gastric isolates were added to AGS gastric epithelial cells (Figure 1C), and intestinal isolates were added to intestinal epithelial Caco-2 cells (Figure 1D). Bacterial isolates from the cervix were added to cervical ME180 cells and are shown in Figure 1C.

Interestingly, the majority of strains tested were able to upregulate EGR1 irrespective of their pathogenicity or Gram staining type (Figures 1A–D). *N. meningitidis* strains of different serogroups, *P. aeruginosa*, as well as the non-pathogenic *N. lactamica* and *N. subflava* all triggered EGR1 expression at 2 h post-inoculation (Figure 1A). A similar induction of EGR1 occurred at 2 h post-inoculation with different serogroups of *S. pyogenes, S. aureus*, and the non-pathogenic oral isolates *L. reuteri* and *L. salivarius* (Figure 1B). The gastric pathogen *H. pylori* triggered EGR1 upregulation at 2 h after infection, whereas the non-pathogenic gastric isolate *L. rhamnosus* did not (Figure 1C). None of the tested *E. coli* strains induced EGR1 in intestinal Caco-2 cells (Figure 1D). *Salmonella enterica* serovar Enteritidis triggered EGR1, whereas *S. enterica* serovar Typhimurium did not (Figure 1D). Of the cervical isolates *N. gonorrhoeae*, but not *L. crispatus*, could induce the transcription of EGR1 (Figure 1C). Western blot analysis showed that the increase in EGR1 expression also occurred at protein level (Figures 1E–H). EGR1 is an early response transcription factor that can be induced rapidly. We detected EGR1 upregulation at 1 h post-inoculation for several strains and a peak in its transcriptional activity at 2 h. Only *Neisseria gonorrhoeae* displayed different time kinetics with the highest EGR1 mRNA levels at 4 h after infection (Figure 1C).

We also examined the gene expression levels of factors that are known to be involved in either the upstream [β1-integrins, epidermal growth factor receptor (EGFR)] or downstream (fibronectin, amphiregulin) signaling of EGR1. No change was detected for β1-integrins and fibronectin (Supplementary Figure S1). Upregulation of the transcript levels of EGFR was observed only upon an infection with *H. pylori* (Supplementary Figure S1). The expression of amphiregulin was induced by a few bacteria, i.e., *P. aeruginosa, S. pyogenes* M5, *N. gonorrhoea* and *L. crispatus*, but not by all bacteria that upregulated EGR1 (Supplementary Figure S1). Moreover, the induction of EGFR and amphiregulin occurred at later time points than that for EGR1, i.e., at 4–6 h post infection. This result suggests that the upregulation of EGFR and amphiregulin are bacterial species specific and independent of EGR1 induction.

We hypothesized that difference in EGR1 induction might be dependent on the amount of bacteria in contact with the host cells. We therefore determined the level of adhesion for each bacterial strain (Figures 1I–L). However, we could not find any correlation between attachment and EGR1 upregulation. For example, *S. enterica* serovar Enteritidis and *S. enterica* serovar Typhimurium adhered to the host epithelial cells at similar levels, but only *S. Enteritidis* induced EGR1 whereas *S. Typhimurium* did not (Figures 1D,L).

Taken together, EGR1 expression can be induced at both the transcriptional and protein level by several species of bacteria and is independent of the level of bacterial adherence, Gram-staining type and pathogenicity.

### Upregulation of EGR1 is cell type specific

**Figure 2:**
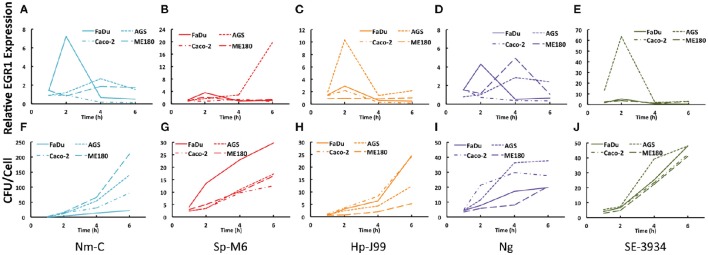
**Cell type specificity in the bacteria-mediated induction of EGR1. (A–E)** Cell line specific induction of EGR1 was investigated during bacterial infection of epithelial cell lines of a pharyngeal (FaDu), gastric (AGS), intestinal (Caco-2), and cervical origin (ME180). Expression of EGR1 was monitored using qPCR at 1, 2, 4, and 6 h post infection. **(F–J)** Bacterial attachment levels to epithelial cells at different time points. Bacteria were diluted and plated for viable counts to determine CFU/ml. Bacteria were added at a MOI of 100 for all experiments. Cells were infected with *N. meningitidis* (Nm-C), *S. pyogenes* (Sp-M6), *H. pylori* (Hp-J99), *N. gonorrhoeae* (Ng) or *S. Enteritidis* (SE-3934).

Different bacteria colonize different sites within the human body. To determine the role of site specificity in the upregulation of EGR1 several cell lines representing different body sites were infected by the same bacterial species. We selected a representative strain from each group; *N. meningitidis* serogroup C (Nm-C), *S. pyogenes* serogroup M6 (Sp-M6), *H. pylori* J99 (Hp-J99), *N. gonorrheae* (Ng), and *S. enterica* serovar Enteritidis (SE-3934). Remarkably, most bacteria could induce the strongest EGR1 response in the cell type of their natural niche and upregulation was low or absent in host cells that did not represent the natural colonization site (Figures 2A–E). An exception is the intestinal pathogen *S. Enteritidis* that induced a very strong response in the gastric epithelial cell line AGS (Figure 2E). Upregulation in other cell types sometimes showed different time kinetics, such as that observed for *N. meningitidis* where EGR1 levels peaked at 2 h in FaDu cells, which represents the natural colonization site of the nasopharynx, EGR1 expression peaked at 4h in ME180, a cervical cell line, and in the gastric epithelial cell line AGS (Figure 2A). Interestingly, infection with *N. gonorrhoeae* showed the same pattern (Figure 2D). This finding is not surprising, because these bacteria are closely related species that colonize both the pharynx and urogenital tract and therefore are likely to induce similar host responses. At 2 h post infection, *S. pyogenes* specifically triggered EGR1 expression in target FaDu cells, whereas after 6 h, EGR1 was unexpectedly induced in AGS cells (Figure 2B).

Similar to the screening experiments, we compared the binding of the bacteria to the different cell lines (Figures 2F–J). Again, no correlation between EGR1 upregulation and adherence could be established. This result was illustrated by *H. pylori*, which adhered equally to all cell types at early time points, but induced different EGR1 expression levels (Figures 2C,H).

Because we detected upregulation of EGFR and amphiregulin for some bacterial strains in initial screening experiments, we also investigated the expression of these genes in the different cell lines (Supplementary Figure S2). Amphiregulin was upregulated by *N. gonorrhoeae* and *S. Enteritidis* in ME180 cells, and some induction could also be detected in Caco-2 cells for *S. Enteritidis* (Supplementary Figure S2). Similarly, EGFR was induced only by *H. pylori* and most pronounced in AGS cells (Supplementary Figure S2). These results strengthen the findings of the screening experiments, which showed that the induction of EGFR and amphiregulin are cell type and species specific, and are possibly not related to EGR1 upregulation.

In conclusion, bacteria-mediated induction of EGR1 is mostly cell type specific and a precise match between bacterium and host cell is necessary to elicit the maximum response.

### Induction of EGR1 is dependent on bacterial viability and contact with the host

**Figure 3:**
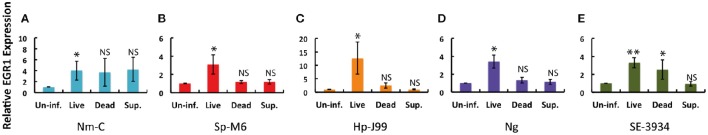
**Bacterial viability and direct contact between bacteria and the host cells affect upregulation of EGR1**. Induction of EGR1 was monitored by qPCR after infection of the host epithelial cells with live or dead bacteria. Dead bacteria were obtained by heat treatment at 95°C for 10 min. Viable counts were used to ensure complete killing of the bacteria. The role of bacterial contact with the epithelial cells in the EGR1 induction was studied using a 0.4 μm Millicell filter, which helps to physically separate the bacteria and host cells, but still allows diffusion of secreted factors in the cell growth media (Sup.). **(A)** Pharyngeal FaDu cells infected with *N. meningitidis* (Nm-C). **(B)** Pharyngeal FaDu cells infected with *S. pyogenes* (Sp-M6). **(C)** Gastric AGS cells infected with *H. pylori* (Hp-J99). **(D)** Cervical ME180 cells infected with *N. gonorrhoeae* (Ng). **(E)** Intestinal Caco-2 cells infected with *S. Enteritidis* (SE3439). Bacteria were added to a MOI of 100 in all experiments. The data was analyzed at 2 h post infection for Nm-C, Sp-M6, SE3439, and Hp-J99. The data was analyzed at 4 h post infection of Ng.

We hypothesized that active interaction between bacteria and host cells was necessary to trigger EGR1 induction. To address this, we infected host epithelial cells with live, heat-killed or live bacteria separated from the host cells by a filter. The maximum induction in the EGR1 was observed with the live bacteria (Figure 3). Heat-killed *N. meningitidis, S. pyogenes, H. pylori* and *N. gonorrhoeae* were not able to induce EGR1, suggesting that bacterial viability is important (Figures 3A–D). However, *S. Enteritidis* could upregulate EGR1 after heat treatment (Figure 3E). After the heat treatment, the bacteria were not washed indicating a possible role of surface molecule or component that is released upon heat treatment might be involved in the upregulation of EGR1 by heat-killed *S*. Enteritidis. Separation of the bacteria from host cells by using a Millicell 0.4 μm filter inhibited EGR1 induction in cells infected with *N. meningitidis*, S. *pyogenes, H. pylori, N. gonorrhoeae*, and *S*. *Enteritidis*. This observation indicates that contact between bacterium and the host cell is necessary (Figure 3).

The data indicate that upregulation of EGR1 in host epithelial cells is dependent on the viability of the bacteria and is usually mediated through contact between the bacterium and host cell.

### Bacteria induce EGR1 through the EGFR–ERK1/2 pathway

**Figure 4:**
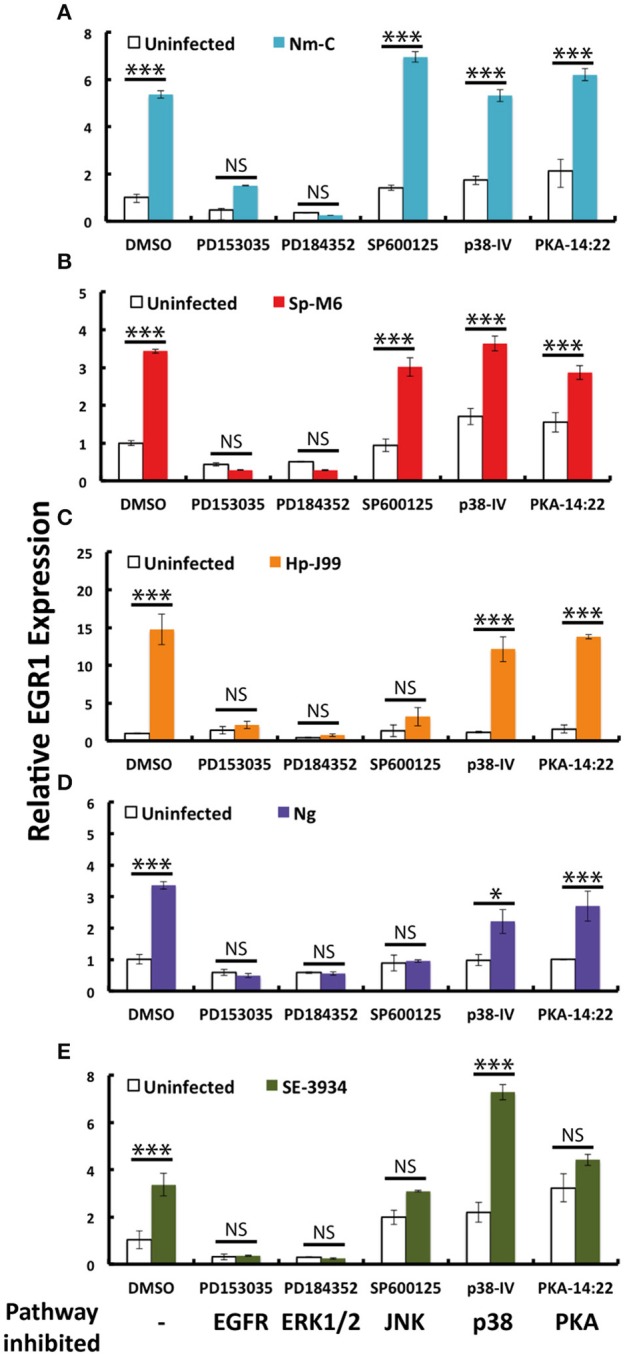
**EGR1 is primarily activated by the EGFR–ERK1/2 pathway upon bacterial infection**. Host epithelial cells were pretreated with PD153035, PD184352, SP600125, P38-IV and PKA-14:22 (inhibiting EGFR, ERK1/2, JNK, p38 and PKA, respectively) 1 h prior to infection. Bacterial infection of the host epithelial cells was carried out by co-incubation with the inhibitors for 2 h, except infection with *N. gonorrhoeae* that continued for 4 h. The expression of EGR1 was analyzed by qPCR. Expression of cells treated with DMSO was set to 1. **(A)** FaDu infected with *N. meningitidis* serogroup C (Nm-C). **(B)** FaDu infected with *S. pyogenes* serogroup M6 (Sp-M6). **(C)** AGS infected with *H. pylori* J99 (Hp-J99). **(D)** ME180 infected with *N. gonorrhoea* MS11 (Ng). **(E)** Caco-2 infected with *S. Enteritidis* (SE-3934). Bacteria were added to a MOI of 100 in all experiments. The white bars represent uninfected controls. The colored bars represent the infected samples. The significant difference between the infected control (DMSO) and the infected samples is marked with asterisk.

Next, we aimed to identify the molecular mechanisms through which bacteria induce EGR1. There are several signaling pathways upstream of EGR1 and therefore we used different chemical inhibitors to specifically block each of these pathways. Interestingly, inhibition of ERK1/2 and EGFR completely abolished the EGR1 upregulation for all the 5 strains tested (Figures 4A–E). Inhibition of signaling through JNK reduced EGR1 upregulation for *H. pylori, N. gonorrhoeae* and *S. Enteritidis* (Figures 4C–E). Inhibition of PKA resulted in a reduction of EGR1 induction specifically for *S. Enteritidis* (Figure 4E). P38 pathway played no role in the bacteria mediated induction of EGR1. Treatment of the host cells with the inhibitors did not affect the adhesion of the bacteria, indicating that EGR1 upregulation is not important for bacterial attachment to host cells (Supplementary Figure S3 online). Since, the blocking of EGFR and ERK1/2 exhibited the strongest inhibition of EGR1 upregulation for all the bacteria tested, we propose that the EGFR–ERK1/2 signaling pathway is a commonly used route for bacterial induction of EGR1.

### Integrin signaling is important for EGR1 upregulation

**Figure 5:**
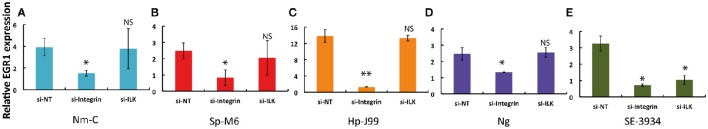
**Integrin mediated signaling in bacteria induced EGR1 expression**. The host epithelial cells were transfected with control siRNA (si-NT), siRNA targeted against β1-integrin (si-Integrin) or directed against integrin-linked kinase (si-ILK) for 60–68 h. The cells were then infected with bacteria with a MOI of 100 for 2 h, except the infection with *N. gonorrhoeae* that continued for 4 h. EGR1 expression was analyzed by qPCR. The graphs represent fold difference in EGR1 expression between infected and uninfected epithelial cells. **(A)** FaDu infected with *N. meningitidis* serogroup C (Nm-C). **(B)** FaDu infected with *S. pyogenes* serogroup M6 (Sp-M6). **(C)** MKN45 infected with *H. pylori* J99 (Hp-J99). **(D)** ME180 infected with *N. gonorrhoea* MS11 (Ng). **(E)** Caco-2 infected with *S. Enteritidis* (SE-3934).

Growth factor receptors can cooperate with integrins to induce signaling and/or enhance the response upon activation by their ligands (Giancotti and Tarone, 2003). Integrin and EGFR cross-talk has also been implicated in EGR1 expression (Cabodi et al., 2009). We therefore used siRNA to silence β1-integrins and integrin-linked kinase (ILK) in host epithelial cells to investigate whether integrin signaling is involved in EGR1 upregulation by bacteria. Silencing β1-integrins completely inhibited the induction of EGR1 expression after infection with *N. meningitidis, S. pyogenes, H. pylori, N. gonorrhoeae*, and *S. Enteritidis* (Figures 5A–E). ILK played role in the upregulation of EGR1 only for *S. Enteritidis* (Figure 5E). The adherence level of bacteria to host cells was not affected by silencing of EGR1, β1-integrins or ILK (Supplementary Figure S4). Downregulation of the transcription of the target genes by siRNA treatment in each cell line was confirmed using qPCR (Supplementary Figure S5). Therefore, signaling through integrins is important for bacteria-mediated EGR1 induction.

## Discussion

The human respiratory, digestive and urogenital tracts are the prime sites of bacterial colonization. Together they offer an area of 300–400 square meters constituting major sites for bacterial adherence (Ribet and Cossart, 2015). Therefore, the present study used epithelial cell lines originating from pharyngeal, gastric, intestinal and cervical tissues and 25 different bacterial strains to study the early host response upon bacterial colonization.

**Figure 6:**
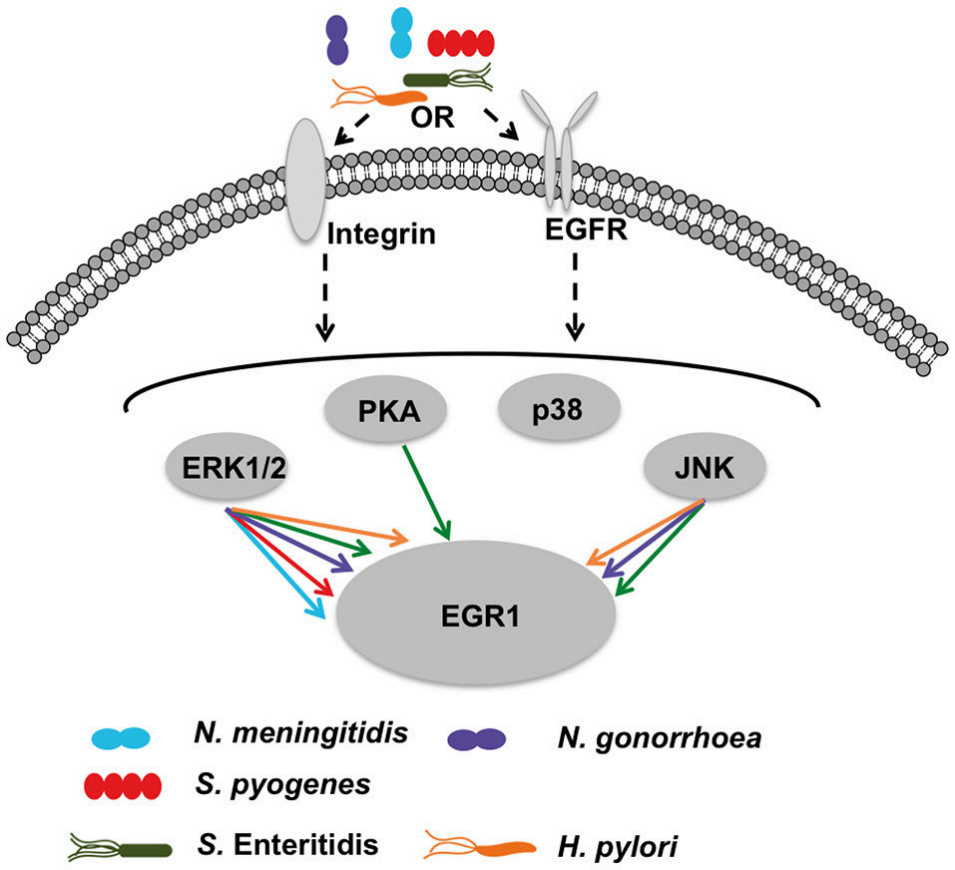
**Hypothetical model of bacteria-mediated EGR1 induction in host epithelial cells**. At the host cell surface, the bacteria may stimulate β1-integrin and/or EGFR. All strains tested exhibit absolute requirement for β1-integrin expression to trigger EGR1 induction (shown in Figure 5). Inhibition of EGFR by PD153035 blocked induction of EGR1 by all strains (shown in Figure 4). Thereby, suggesting that activation of EGFR through β1-integrin as a possible mechanism for bacteria mediated induction of EGR1. Bacterial contact with the host cells was required for the induction of EGR1 response (shown in Figure 3). Integrins and EGFR can activate several signaling molecules inside the host cell that can in turn lead to induction of EGR1. Signaling through ERK1/2 was critical for all strains, since the induction of EGR1 was blocked by ERK1/2 inhibitor (Figure 4). Also other signaling factors are partially involved in a species-dependent manner. Data suggest that *H. pylori, N. gonorrhoeae* and *S. Enteritidis* can signal through JNK to upregulate EGR1, whereas PKA is utilized only by *S. Enteritidis*.

EGR1 is an early response transcription factor that can be induced by different stimuli. Here, we show that several strains of bacteria upregulate EGR1 expression in epithelial cells during the initial colonization of the host. For all the bacterial strains that induced EGR1 expression the maximal induction was observed at 2 h except for *N. gonorrhoea* occurring at 4 h. The host response is independent of adhesion levels, Gram-staining type and pathogenicity of the bacteria, but dependent on host cell contact and bacterial viability. In the assays to examine the role of cell type specificity in the upregulation of EGR1 it was observed that EGR1 was induced at different levels. However, the strongest response in the induction of EGR1 was mainly observed in the epithelial cells originating from the natural colonization site of the bacteria, indicating that the process is cell type specific. Using chemical inhibitors and RNA silencing we were able to identify β1-integrins, EGFR and ERK1/2 as the main signaling molecules mediating the EGR1 upregulation by bacteria. However, for some bacterial strains a possible role for other host signaling pathways was observed (Figure 6).

Many bacteria have been shown to associate with integrins (Hauck et al., 2012). Integrins can induce EGR1 expression through different signaling pathways (Lee et al., 2008; Cabodi et al., 2009). Here, we demonstrate for the first time that β1-integrins are often necessary for the bacterial induction of EGR1. Several bacteria have been shown to activate the EGFR, including *N. menigitidis, N. gonorroeae, H. pylori, S. Typhimurium* and *P. aeruginosa*. Often, this activation is indirect and mediated by bacterial induction of the cleavage of EGFR ligands by metalloproteases, allowing the ligands to bind to and activate EGFR (Keates et al., 2001; Zhang et al., 2004; Slanina et al., 2014). The importance of EGFR for EGR1 upregulation has been demonstrated in several studies (Meagawa et al., 2009; Voena et al., 2013). One study has shown the involvement of EGFR in the induction of EGR1 by a bacterial stimulus, *H. pylori* (Keates et al., 2005). Here, we confirm the importance of EGFR in EGR1 regulation and additionally demonstrate that EGFR is a common signaling molecule in bacteria-mediated EGR1 induction. Further, our data shows that ERK1/2 is another common signaling molecule in this process, but that EGR1 can also occasionally be induced by the JNK and PKA pathways.

Crosstalk between integrins and growth factor receptors has been extensively studied and can be bidirectional (Giancotti and Tarone, 2003). Cabodi et al. showed that integrin-EGFR complex formation is necessary for EGR1 expression that is induced by integrin-mediated adhesion (Cabodi et al., 2009). Inhibition of β1-integrin caused significant (*p* < 0.05) reduction in the induction of EGR1 by *N. meningitidis, N. gonorrhoeae, S. pyogenes, H. pylori*, and *S*. *Enteritidis*. In addition, the inhibition of EGFR completely abrogated the induction of EGR1 by *N. meningitidis, N. gonorrhoeae, S. pyogenes, H. pylori*, and *S*. *Enteritidis*.

In addition to the bacterial species described in the literature, we show that several other bacterial strains can upregulate EGR1 expression. EGR1 induction seems to be a general response of epithelial cells to bacterial colonization. In addition, we observed cell type specificity and variation in the signaling pathways used by different bacteria. Therefore, it is unlikely that EGR1 is induced by one molecule that is conserved among bacteria. Furthermore, heat killed *S*. *Enteritidis* was sufficient for EGR1 induction whereas other bacteria show requirement for viability and direct contact with the host cell. However, the role of surface molecule or bacterial component released in the supernatant upon heat treatment of *S*. *Enteritidis* needs further investigation. This result indicates a difference in the nature of bacterial EGR1 inducing factors. There are some indications in literature on the type of components could be involved, which represent a variety of molecules. For example, the type IV secretion system encoded by the Cag pathogenicity island is required for EGR1 induction by *H. pylori* (Keates et al., 2005). *S. aureus* can upregulate EGR1 through peptidoglycan (Xu et al., 2001). The molecule that is required by the bacteria also most likely depends on the host cell type. EGR1 upregulation by *S. Typhimurium* SL1344 and enteropathogenic *E. coli* in epithelial cells is dependent on their type III secretion systems (de Grado et al., 2001; Hannemann et al., 2013), whereas LPS from *E. coli* and *Salmonella minnesota* can induce EGR1 in monocytes (Coleman et al., 1992; Guha and Mackman, 2002).

EGR1 is a transcription factor with many downstream targets. It has been shown that EGR1 can bind to the promotors of several proteins involved in inflammation, such as the pro-inflammatory cytokines IL6, IL8 and TNF, and stimulate their expression (Shi et al., 2002; Droin et al., 2003; Hoffmann et al., 2008; Ma et al., 2009; Lin et al., 2014). In this way, EGR1 could help the host to mount an initial defense against invading pathogens.

Among the targets of EGR1 are many genes that are involved in proliferation and prevention of apoptosis, which gives EGR1 a putative role in the development of cancer. Indeed, EGR1 is overexpressed in certain cancers, such as prostate cancer, gastric cancer and cervical cancer (Eid et al., 1998; Akutagawa et al., 2008; Zheng et al., 2010). Bacterial infections have been linked to cancer, mainly through epidemiological studies. The most widely accepted link is between *H. pylori*, which was the first bacterium to be declared as a carcinogen, and gastric cancer (Sokic-Milutinovic et al., 2015). However, other associations have been made, such as *S. enterica* as a causative agent of gallbladder cancer (Scanu et al., 2015). The upregulation of EGR1 upon bacterial infection is therefore a possible important event in bacteria-associated cancer development.

EGR1 can be upregulated by bacteria in host cells, and ERK has often been identified as a key signaling molecule in this process. The study has certain limitations imposed by the adopted experiments for adhesion assays. The bacteria were not removed after adding to the epithelial cells. Therefore, during the incubation period, the bacteria could grow and alter the composition of the medium with respect to nutrient composition and release of metabolites. The study has certain limitations imposed by the adopted experiments for adhesion assays. The bacteria were not removed after adding to the epithelial cells. Therefore, during the incubation period, the bacteria could grow and alter the composition of the medium with respect to nutrient composition and release of metabolites. However, both heat-killed and live *S. Typhimurium* were able to induce EGR1 at 2 h post infection (Figure 3E). Also, cell type specificity in the bacteria-mediated induction of EGR1 was observed in different cell lines (Figure 2). These observations indicated that bacteria-mediated EGR1 induction might not be due to alterations in the composition of the growth media. Instead, there is a possible role of an interaction between the bacteria and host in the bacteria-mediated induction of EGR1. Identification of the bacterial component(s) that induce EGR1 signaling and the consequences of the EGR1 induction toward bacterial pathogenicity or host defense require further investigation. The present study shows how widespread the EGR1 response is among bacteria and adds EGFR and integrin signaling as important contributors to EGR1 induction.

## Author contributions

NdK and SS contributed equally to the manuscript. Conceived and designed the experiments: NdK, SS, LM, and A-BJ. Performed experiments: NdK, SS, and GW. Analyzed the data and wrote manuscript: NdK, SS, and A-BJ.

## Funding

This work was supported by the Swedish Research Council (Dnr 2006-4112, 2012-2415, 2013-2434), The Swedish Cancer Society, and Torsten Söderbergs Stiftelse.

### Conflict of interest statement

The authors declare that the research was conducted in the absence of any commercial or financial relationships that could be construed as a potential conflict of interest.
