# Supplementary material for: The Host Cell Transcription Factor EGR1 Is Induced by Bacteria through the EGFR–ERK1/2 Pathway
Source: Front Cell Infect Microbiol. 2017 Jan 25;7:16. doi: 10.3389/fcimb.2017.00016 (PMC5264520; doi:10.3389/fcimb.2017.00016)
Supplement: Supplementary file 1 [file Image1.PDF]

Supplementary Information

The host cell transcription factor EGR1 is induced by bacteria through the EGFR - ERK1/2 pathway

Nele de Klerk[#], Sunil D. Saroj[#], Lisa Maudsdotter, Gabriela M. Wassing, Ann-Beth Jonsson[*]

Department of Molecular Biosciences, The Wenner-Gren Institute, Stockholm University, Stockholm, Sweden

[#] **These authors contributed equally**

[*] **Corresponding author**

Ann-Beth Jonsson, E-mail: ann-beth.jonsson@su.se, Phone: +46 8 164154

Supplementary figure S1

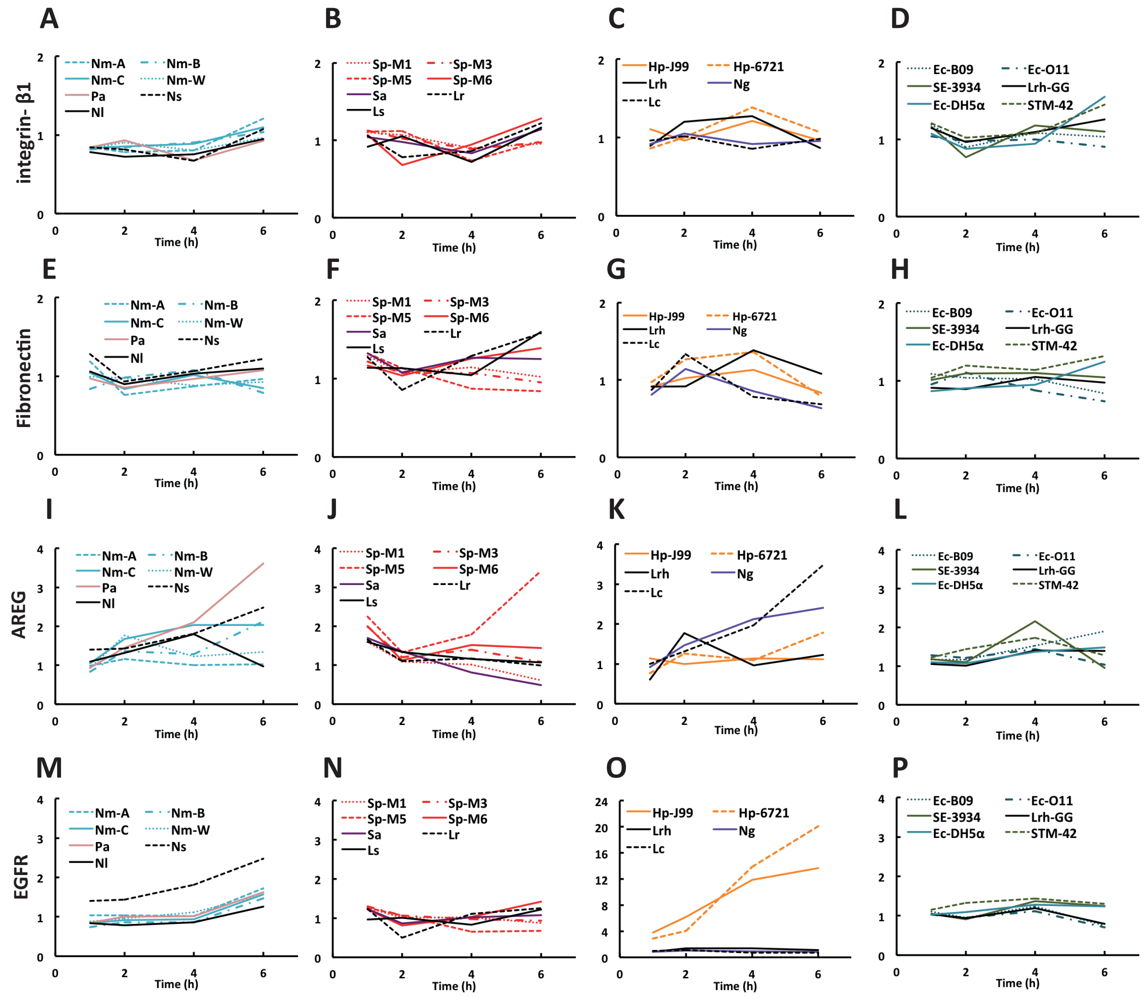

Figure S1. Expression of β1-integrin, fibronectin, amphiregulin and EGFR genes in epithelial cells upon bacterial infection.

Epithelial cell lines were infected with bacteria described in Table 1. Upregulation of β1-integrin (a-d), fibronectin (e-h), amphiregulin (AREG) (i-l) and EGFR (m-p) were monitored at the transcriptional level using qPCR at 1, 2, 4 and 6 h post inoculation. (a, e, i, m) Pharyngeal FaDu cells inoculated with different strains of *N. meningitidis* (Nm-A, Nm-B, Nm-C, Nm-W), *P. aeruginosa* (Pa), *N. subflava* (Ns), and *N. lactamica* (Nl). (b, f, j, n) FaDu cells inoculated with *S. pyogenes* (Sp-M1, Sp-M3, Sp-M5, Sp-M6), *S. aureus* (Sa), *L. reuteri* (Lr), and *L. salivarius* (Ls). (c, g, k, o) Gastric AGS cells inoculated with *H. pylori* (Hp-J99, Hp-6721) and *L. rhamnosus* (Lrh). Cervical ME-180 cells infected with *N. gonorrhoeae* (Ng) and *L. crispatus* (Lc). (d, h, l, p) Intestinal Caco-2 cells inoculated with *E. coli* (Ec-B09. Ec-O11, Ec-DH5α), *L. rhamnosus* GG (Lrh-GG), *Salmonella enterica* serovar Enteritidis (SE-3934) and *Salmonella enterica* serovar Typhimurium (STM-42).

Supplementary figure S2

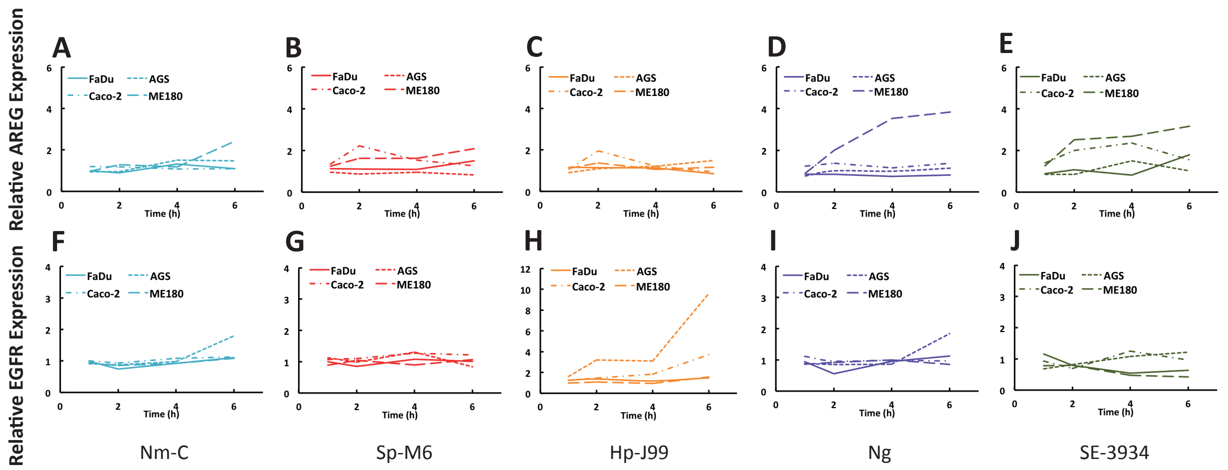

Figure S2. Cell type specificity in the expression of amphiregulin and EGFR genes in epithelial cells upon bacterial infection.

Cell line specific induction of amphiregulin (AREG) a-e) and EGFR (f-j) was studied by bacterial infection of epithelial cell lines from pharyngeal (FaDu), gastric (AGS), intestinal (Caco-2) and cervical origin (ME180). Transcriptional activity was monitored by qPCR at 1, 2, 4, and 6 h post infection. Bacteria were added to a MOI of 100 in all experiments. Cells were infected with *N. meningitidis* (Nm-C), *S. pyogenes* (Sp-M6), *H. pylori* (Hp-J99), *N. gonorrhoeae* (Ng) or *S*. Enteritidis (SE-3934).

Supplementary figure S3

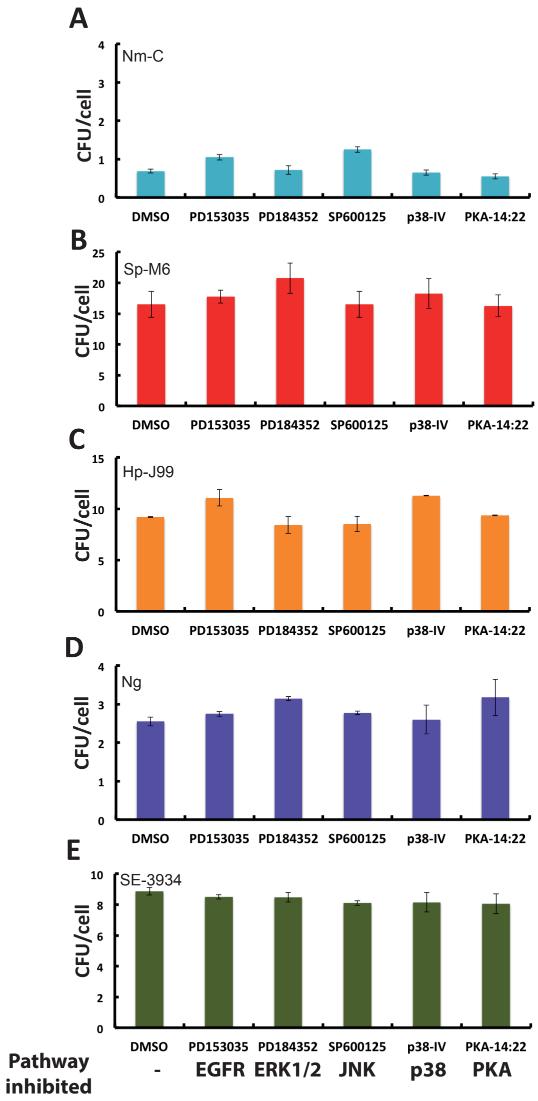

Figure S3. Role of EGR1 upstream signaling in bacterial adhesion.
Host epithelial cells were pretreated with PD153035, PD184352, SP600125, P38-IV and PKA-14:22 (inhibiting EGFR, ERK1/2, JNK, p38 and PKA, respectively) 1 h prior to infection. Bacterial infection of the host epithelial cells was carried out by co-incubation with the inhibitors for 2 h, except infection with *N. gonorrhoeae* that continued for 4 h. The graphs represent bacterial adhesion to the host epithelial cells as determined by viable counts. (a) FaDu infected with *N. meningitidis* serogroup C (Nm-C). (b) FaDu infected with *S. pyogenes* serogroup M6 (Sp-M6). (c) AGS infected with *H. pylori* J99 (Hp-J99). (d) ME180 infected with N. gonorrhoea MS11 (Ng). (e) Caco-2 infected with S. Enteritidis (SE3439). Bacteria were added to a MOI of 100 in all experiments. Inhibition of the pathways upstream of EGR1did not exhibit significant (P>0.05) difference in the bacterial adhesion.

Supplementary figure S4

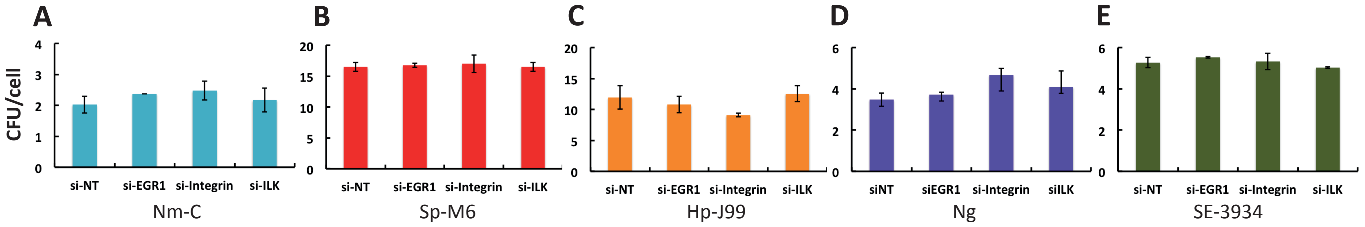

Figure S4. The role of EGR1 and integrin signaling in bacterial adhesion.

The host epithelial cells were transfected with control siRNA (si-NT), siRNA targeted against β1-integrin (si-Integrin) or directed against interin-linked kinase (si-ILK) for 60-68 h. The cells were then infected with bacteria with a MOI of 100 for 2 h, except the infection with *N. gonorrhoeae* that continued for 4 h. The graphs represent bacterial adhesion to the host epithelial cells as determined by viable counts. (a) FaDu infected with *N. meningitidis* serogroup C (Nm-C). (b) FaDu infected with *S. pyogenes* serogroup M6 (Sp-M6). (c) AGS infected with *H. pylori* J99 (Hp-J99). (d) ME180 infected with *N. gonorrhoea* MS11 (Ng). (e) Caco-2 infected with *S.* Enteritidis (SE3439). The inhibition of EGR1, integrin or ILK had no significant (P>0.05) effect on the bacterial adhesion.

Supplementary figure S5

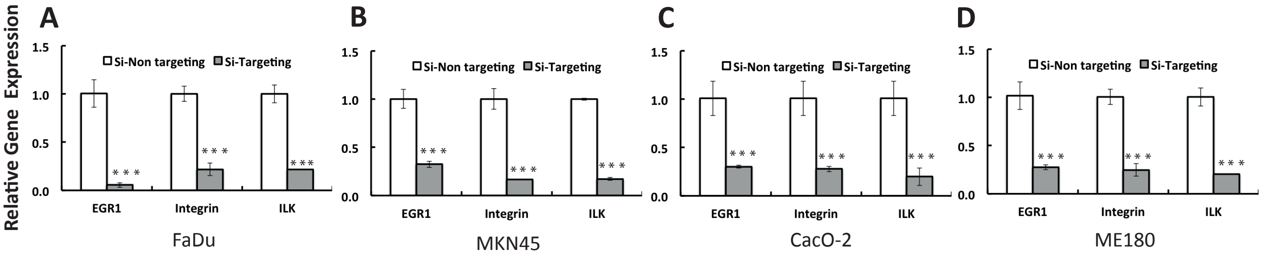

Figure S5. Detection of siRNA induced gene silencing.

The host epithelial cells were transfected with the indicated siRNA for 60-68 h. Post transfection cells were harvested and assayed for expression of the specific target genes by qPCR.
